# A Comprehensive Review on Equine Influenza Virus: Etiology, Epidemiology, Pathobiology, Advances in Developing Diagnostics, Vaccines, and Control Strategies

**DOI:** 10.3389/fmicb.2018.01941

**Published:** 2018-09-06

**Authors:** Raj K. Singh, Kuldeep Dhama, Kumaragurubaran Karthik, Rekha Khandia, Ashok Munjal, Sandip K. Khurana, Sandip Chakraborty, Yashpal S. Malik, Nitin Virmani, Rajendra Singh, Bhupendra N. Tripathi, Muhammad Munir, Johannes H. van der Kolk

**Affiliations:** ^1^ICAR-Indian Veterinary Research Institute, Bareilly, India; ^2^Division of Pathology, ICAR-Indian Veterinary Research Institute, Bareilly, India; ^3^Central University Laboratory, Tamil Nadu Veterinary and Animal Sciences University, Chennai, India; ^4^Department of Biochemistry and Genetics, Barkatullah University, Bhopal, India; ^5^National Research Centre on Equines, Hisar, India; ^6^Department of Veterinary Microbiology, College of Veterinary Sciences and Animal Husbandry, West Tripura, India; ^7^Division of Biological Standardization, ICAR-Indian Veterinary Research Institute, Bareilly, India; ^8^Division of Biomedical and Life Sciences, Lancaster University, Lancaster, United Kingdom; ^9^Division of Clinical Veterinary Medicine, Swiss Institute for Equine Medicine (ISME), Vetsuisse Faculty, University of Bern and Agroscope, Bern, Switzerland

**Keywords:** equine, influenza virus, epidemiology, pathogenesis, diagnosis, vaccine, prevention, control

## Abstract

Among all the emerging and re-emerging animal diseases, influenza group is the prototype member associated with severe respiratory infections in wide host species. Wherein, Equine influenza (EI) is the main cause of respiratory illness in equines across globe and is caused by equine influenza A virus (EIV-A) which has impacted the equine industry internationally due to high morbidity and marginal morality. The virus transmits easily by direct contact and inhalation making its spread global and leaving only limited areas untouched. Hitherto reports confirm that this virus crosses the species barriers and found to affect canines and few other animal species (cat and camel). EIV is continuously evolving with changes at the amino acid level wreaking the control program a tedious task. Until now, no natural EI origin infections have been reported explicitly in humans. Recent advances in the diagnostics have led to efficient surveillance and rapid detection of EIV infections at the onset of outbreaks. Incessant surveillance programs will aid in opting a better control strategy for this virus by updating the circulating vaccine strains. Recurrent vaccination failures against this virus due to antigenic drift and shift have been disappointing, however better understanding of the virus pathogenesis would make it easier to design effective vaccines predominantly targeting the conserved epitopes (HA glycoprotein). Additionally, the cold adapted and canarypox vectored vaccines are proving effective in ceasing the severity of disease. Furthermore, better understanding of its genetics and molecular biology will help in estimating the rate of evolution and occurrence of pandemics in future. Here, we highlight the advances occurred in understanding the etiology, epidemiology and pathobiology of EIV and a special focus is on designing and developing effective diagnostics, vaccines and control strategies for mitigating the emerging menace by EIV.

## Introduction

Equine influenza (EI) is an extremely contagious disease of horses (including wild horses), which is caused by Influenza A viruses. These viruses are known for high rates of transmission in a wide variety of animal species. Equine influenza virus (EIV), the causative agent of EI, is considered to be one of the most important viral respiratory pathogens of equines. The disease is characterized by flu-like symptoms affecting predominantly the respiratory tract (Wilson, 1993; Slater and Hannant, 2000; van Maanen and Cullinane, 2002; Newton and Mumford, 2005; Cullinane et al., 2006; Landolt et al., 2007; OIE, 2008; Stack et al., 2013; Kapoor and Dhama, 2014; Yin et al., 2014). The presence of influenza infections has been suggested in horses since the time of Hippocrates and Absyrtus, the latter being a Greek veterinarian, described a disease resembling influenza in 412 BC and 433 AD, respectively. In 1872, an outbreak of influenza occurred throughout the North America and affected large population of horses resulting in crippled transportation of goods, unloading of ships and stoppage of almost all essential services (Law, 1874). In India, one of the largest EI outbreak occurred in 1987 affecting more than 27,000 equines and causing death of several hundred (Uppal et al., 1989). In Australia during 2007, an EI outbreak infected ~10,000 equines despite keeping strict preventive and control measures. The disease was restricted after great efforts at the colossal cost of about one billion Australian dollars (Cowled et al., 2009).

Equine influenza is mainly caused by two subtypes of influenza A viruses namely H7N7 (first isolated in the year 1956) and H3N8 (first isolated in the year 1963; Sovinova et al., 1958; Waddel et al., 1963). Previously, H7N7 was considered as the major cause of epidemics whereas latter H3N8 strain is mainly responsible for outbreaks across the globe (Mathew et al., 2010; Bryant et al., 2011; Alves Beuttemmüller et al., 2016). Genetic analysis has revealed close relatedness of H3N8 strains of EI with avian influenza virus (AIV), which may indicate co-existence of influenza viruses in aves and equines (Cullinane and Newton, 2013). Notably, EIV has been seen to infect unusual host, dogs (Kirkland et al., 2010; Wang et al., 2017). Although, before 2004, canines were considered resistant to influenza virus infection, the recent epidemic of influenza in canines came as a surprise (Gibbs and Anderson, 2010). Interspecies transmission of the virus has been reported in racing greyhounds in the USA where the isolated virus showed close relatedness to H3N8 virus (Crawford et al., 2005). Further studies confirmed that though this virus was earlier considered to be confined to the equine host exclusively, it has been demonstrated in canines (Crawford et al., 2005; Gibbs and Anderson, 2010; Hayward et al., 2010; Landolt, 2014), zebras, camels (Yondon et al., 2014), and humans (Larson et al., 2015).

The evolution (intra-host) of EIV has been recorded in naive horses and during field outbreaks (Murcia et al., 2010; Hughes et al., 2012). Antigenic drift (caused by point mutation) has resulted in emergence of “European” and “American” lineages of H3N8 (Wilson, 1993; Daly et al., 1996, 2004; Oxburgh and Klingeborn, 1999; Purzycka et al., 2004; Cullinane and Newton, 2013). As of now, human infections with EIV have not been reported, and only serological evidences exists without virus isolation from specimens. Although, zoonotic implications of EIV have not yet been fully elucidated, nevertheless, the virus can pose a threat to laboratory personnel (Alexander and Brown, 2000; OIE, 2008). Since detection of EIV in dogs, it is presumed that this virus can re-assort (H3N8) with human influenza virus and might lead to the emergence of novel strains (Na et al., 2016).

Although vaccination is the most useful prophylactic strategy; continuous genetic evolution of the virus demands genetic characterization of currently circulating EIVs for the selection of a candidate vaccine strain. As vaccine failures occur in several parts of the world there is need for a better vaccine to completely eradicate equine influenza (Kinsley et al., 2016). Hence, understanding of the molecular mechanisms involved with its cross-species transmission is of prime importance to devise any prophylactic and control strategy (Holland, 2003; Smyth, 2007; Joseph et al., 2017).

The present review comprehensively describes EIV and the disease it causes, epidemiology, transmission, pathogenesis and pathology, advances in diagnosis, vaccine development and appropriate prevention, and control strategies to be adapted.

## Etiology

**Figure 1:**
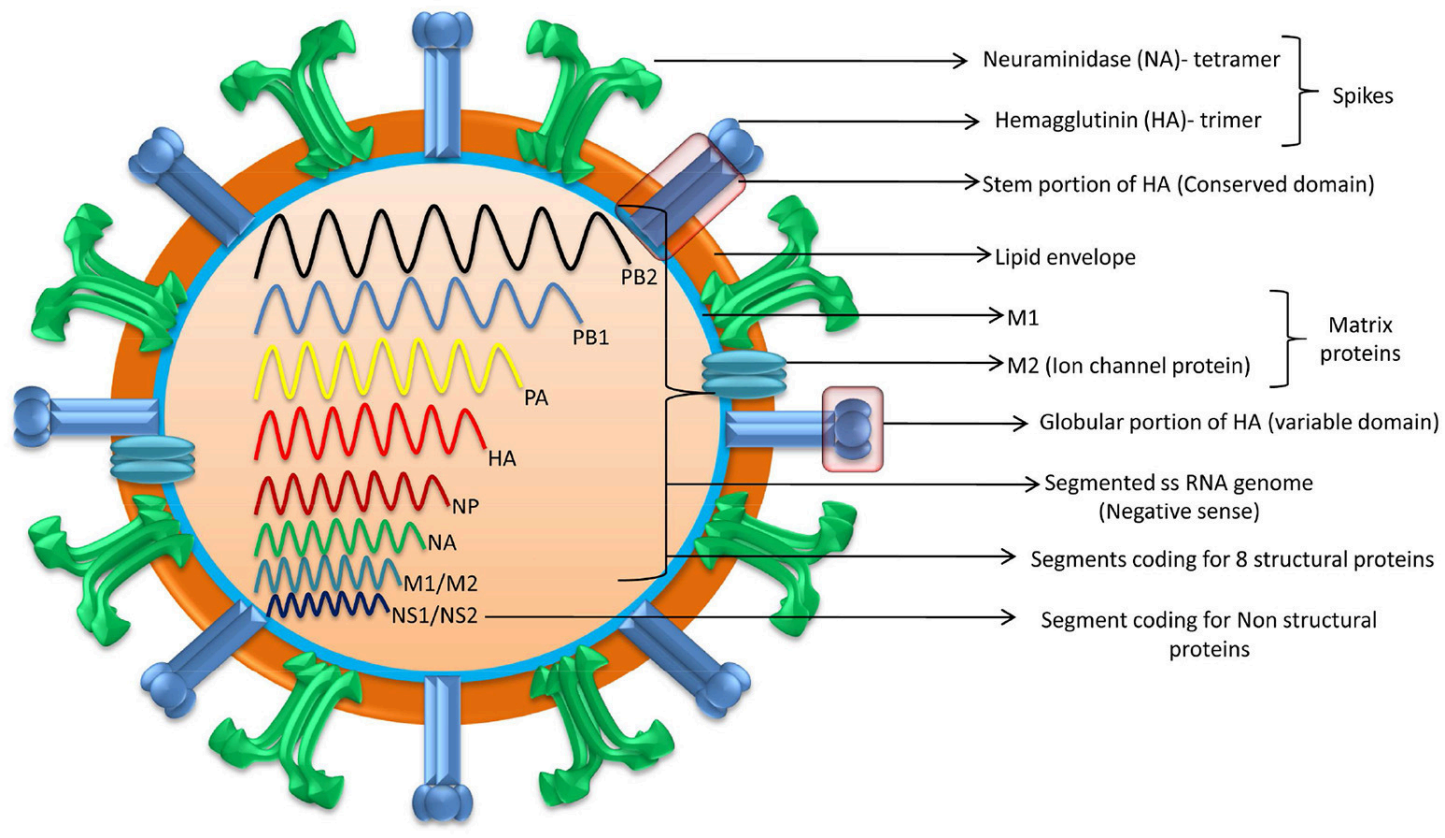
Structure of Equine Influenza Virus. EIV is a segmented RNA virus possessing eight (single) segmented negative sense RNA strands. Segmented genome encodes eight structural proteins and at least two non-structural proteins.

Based on the matrix and nucleoprotein genes of influenza viruses, they have been classified as type A, B, C, and D. Type A viruses mainly infect animals and humans while type B and C viruses infect only humans. A close relative of Type C virus, Type D was first reported in the year 2011 from swine and later identified from cattle, sheep, and goats (Ferguson et al., 2016). Sero-surveys showed that type D antibodies were found in humans and equines also (Nedland et al., 2018). AIV is considered as an ancestor to all other influenza viruses of mammalian and non-mammalian species. H7N7 and H3N8, the major subtypes of EIV, were previously referred as equine 1 and 2 viruses, respectively (Daly et al., 2011; Chambers, 2014; Sreenivasan et al., 2018). EIV is a segmented RNA virus with 80–120 nm in diameter and is classified under the family *Orthomyxoviridae* belonging to the genus *Influenza* A (Timoney, 1996). Influenza A viruses possess eight single segmented negative sense RNA strands and are sub-typed based on the two surface glycoproteins that make 45% of the mass of this virus, namely hemagglutinin (HA) and neuraminidase (NA) (Webby et al., 2007; Cullinane and Newton, 2013; Lewis et al., 2014). The shape of EIV particle is greatly determined by segment 7 (Elton et al., 2013). The segmented genome of EIV encodes at least 10 classical proteins. The proteins that are encoded by the segmented genome are: structural proteins which are termed as HA, NA, nucleoprotein (NP), matrix proteins (M1 and M2), three polymerase proteins (PB1, PB2, and PA), one nuclear export protein (NEP) and a non-structural protein named as NS1. As a result of complementary sequences and frame shift other minor yet important proteins are also expressed. HA and NA glycoproteins are termed as spikes as these projects outside the envelope, essential for viral entry and release (Timoney, 1996; Easterday et al., 1997; Figure 1).

Complete transcription of segment 8 leads to expression of NS1 while pre-mature splicing leads to expression of NEP (Lamb and Lai, 1980). Earlier NEP was thought to be a non-structural protein and termed as NS2 and later studies indicated that this protein was found within virion and have interaction with M protein (Paterson and Fodor, 2012). NEP is essential for the release of viral ribonucleoprotein from the host nucleus. Viral RNA segments 2 and 3 codes for PB1 and PA which are the major virulence factors. Further, PB1 subunit can give rise to three proteins namely, PB1, PB1-F2, and PB1-N40. +1 reading frame of PB1 segment codes for the PB1-F2 (on average 90 amino acid length) which has apoptosis induction function (Krumbholz et al., 2011). N40 is another version of PB1 where there is truncation in the N terminal region of PB1 (Wise et al., 2009). PA-X is a recently described protein which is the outcome of ribosomal frame shifting of segment 3 mRNA during translation (Jagger et al., 2012). It is noteworthy that the PA-X protein of the virus causes suppression of host gene expression (Feng et al., 2016; Oishi et al., 2018).

NS1, a homodimer protein (215–237 amino acids), is an important virulence factor of influenza virus as it modulates several viral and host cellular mechanisms during influenza replication cycle. There are two functional domains in case of NS1 protein named as RNA binding domain (N terminal end) and effector domain (C terminal end; Chien et al., 2004). NS1 possess different epitopes hence having multifunctional activities. NS1 protein plays a crucial role in influenza infection by antagonizing type I interferon of host and reducing IFN β production (Hale et al., 2008). On the basis of nucleotide homology, NS segment of influenza A virus are divided into A and B allele. All mammalian influenza isolates except equine origin H3N8 belong to allele A (Guo et al., 1992).

HA and NA proteins are the important surface antigens in EIC and antibodies generated against them provide resistance to infection (Landolt, 2014). Neutralizing antibodies are formed against HA that can block virus entry and antigenic drift at this molecule can lead to vaccine failure (Yates and Mumford, 2000). Similarly, protective antibodies against NA aggregates the virus on host cell surface and hinders the virus release from the cells (Sylte and Suarez, 2009). Heterotypic immunity is provided at minimum level by humoral responses; whereas cross-reactive response (mediated by cytotoxic T lymphocytes) is observed between the viral subtypes, true for all the subtypes of type A viruses (Hemann et al., 2013; McKinstry et al., 2013; Landolt, 2014). Subtle changes in the constitution of amino acid may result in immune escape due to different antigenicity (Park et al., 2009) and recently, it has been revealed that there are nine substitutions in the sequences of HA of Brazilian EIV isolates in comparison to the vaccine strain (Florida Clade 1; Favaro et al., 2018).

A detailed investigation of 1989 UK outbreak using reverse genetics and site-directed mutagenesis determined the role of amino acid substitutions within HA glycoprotein (Woodward et al., 2015), and mutations at positions 159, 189, and 227 were found to be associated with altered antigenicity, as revealed by HI assays. The antigenic site B was suggested to be the major antigenic site (Daly et al., 1996) and K189 in it is also important for differentiation in Eurasian sub-lineage (Lewis et al., 2011). K189 residue retains a very important role in pertaining antigenicity and switching between uncharged, acidic and basic amino acid is responsible for differed antigenic properties (Ye et al., 2013). The same position has been noticed important for altered antigenic phenotype in H3N2 viruses (Koel et al., 2013), and the same mutation has been found culprit for human vaccine breakdown in Iran during 2005–2006 (Moattari et al., 2010).

## EIV subtypes, lineages, and sublineages

**Figure 2:**
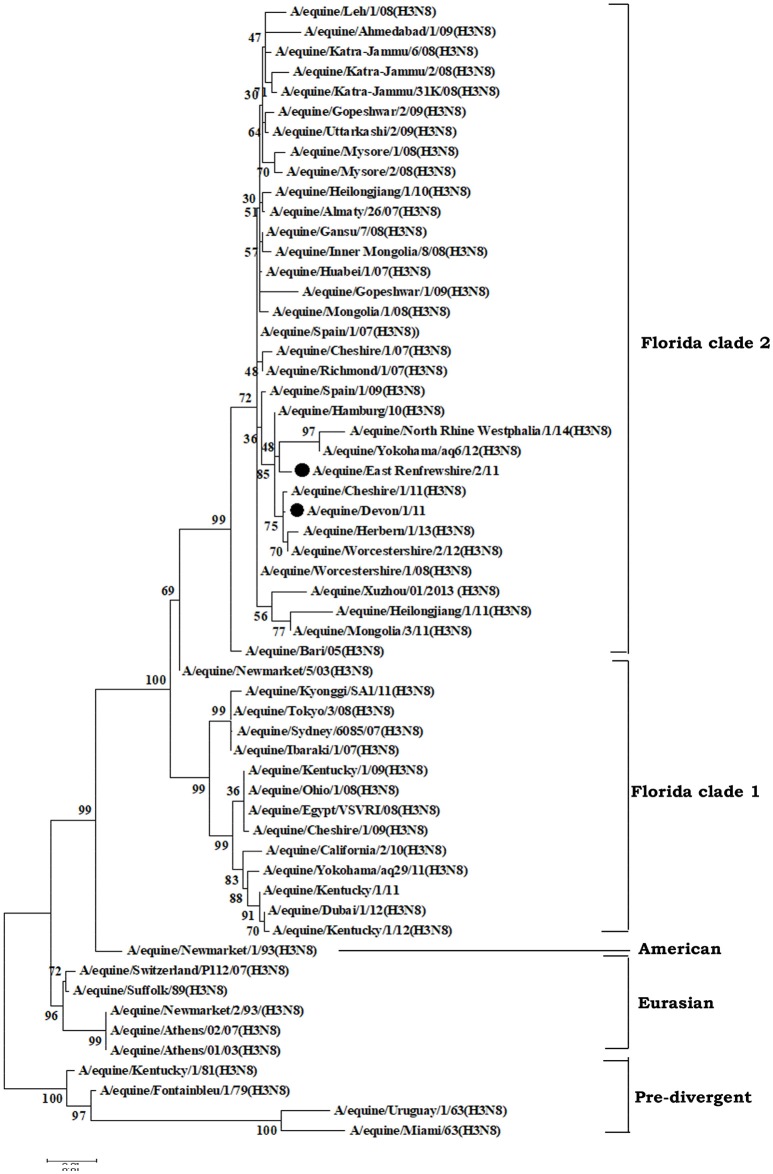
Phylogenetic analysis of hemagglutinin (HA) genes nucleotide sequences from 57 Equine Influenza Viruses (EIVs). The maximum likelihood tree was constructed using stringent T92 + G algorithm which was identified using the find best DNA/protein model tool available in MEGA 6. The reliability of the trees was assessed by bootstrap with 1,000 replications with cut off at 50 are shown in the tree. The phylogram depicts five major clusters of global EIVs. Phylogenetic group's viz., Florida sub-lineage clade 1, Florida sub-lineage clade 2, American, Eurasian and Pre-divergent, are mentioned by bars on the right. The major mutations (I179V and A144V) observed in the Clade 2 viruses of Florida sublineage in recent isolates have been denoted by solid dots.

EIV has two recognized subtypes namely H7N7 (subtype 1) and H3N8 (subtype 2), of which H3N8 predominantly circulates in equines. H3N8 subtype was isolated for the first time in 1963 from the horses showing the symptoms of flu in USA, designated as A/eq/Miami/63 and is considered as prototype virus (Waddel et al., 1963). Earlier, it was hypothesized that H3N8 subtype viruses evolved as a single lineage (Kawaoka et al., 1989). Based on the sequence analysis of the HA gene, the H3N8 EIV shows two genetic and antigenic variants (Figure 2) evolving after 1980s namely Eurasian and American lineages (Daly et al., 1996). Subsequently, American lineage evolved into three sublineages namely Argentinian, Kentucky, and Florida (Lai et al., 2001). Further, evolution of the Florida sublineage has resulted in the emergence of two groups of viruses with divergent HA sequences (Figure 2) which are provisionally referred to as Florida sublineage clades 1 and 2 viruses (Bryant et al., 2011). Currently, Clade 1 and Clade 2 lineage viruses have been circulating across the globe and leading to outbreaks. Clade 1 viruses have been circulating more in American continent while Clade 2 viruses have been incriminated for most of the outbreaks in Europe and Asia. Both clades have been reported in major outbreaks throughout the world (Bryant et al., 2009). However, outbreaks due to both of them keep on occurring across the geographic barriers. Florida Clade 1 viruses have been responsible for major outbreaks in Japan and Australia in 2007–08 (Bryant et al., 2009) while Clade 2 viruses caused huge outbreaks in China, India, and Mongolia (Virmani et al., 2010a). A/eq/South Africa/04/2003-like or A/eq/Ohio/2003-like viruses are representative of clade 1, A/eq/Richmond/1/2007-like viruses represents clade 2 and Newmarket/2/93 represents Eurasian lineage (Laabassi, 2016). Since 2013, some of the isolates from Europe have been consistently showing two amino acid changes *viz*. A144V and I179V (Figure 2) in the antigenic region and have been referred two as subgroups in Clade 2 lineage. The changes are in the antigenic site; however, they have not affected the HI assay using the post-infection Ferret antisera (Woodward et al., 2014; Rash et al., 2017).

## Epidemiology and evolution

Since 1956, when marked widespread respiratory epidemic disease occurred in equines due to EIV, Europe and North America are the most endemic regions for EI, and almost all nations in the world have witnessed outbreaks caused by EIV except few small island countries like New Zealand and Iceland. Currently, EI is prevalent worldwide *viz*. Europe, Canada, USA, Turkey, Scandinavia, and South America (Sovinova et al., 1958; Waddel et al., 1963; Gerber, 1970; Uppal et al., 1987; Wood and Mumford, 1992; Wilson, 1993; Timoney, 1996; van Maanen and Cullinane, 2002; Radostits et al., 2003; Purzycka et al., 2004; Newton and Mumford, 2005; Mathew et al., 2006; Landolt et al., 2007; Smyth, 2007; Foord et al., 2009; Sajid et al., 2013; Gahan et al., 2017). Increase in equine traffic has led to the spread of EIV to other countries including South Africa in 2003. Australia has reported the disease for the first time in 2007 and the disease was re-introduced to Japan and South Africa (Ito et al., 2008; OIE, 2008; Yamanaka et al., 2008a; Foord et al., 2009; Alves Beuttemmüller et al., 2016). Improper quarantine of sub-clinically affected animals, which were not sufficiently vaccinated, led to spread of this virus to Australia as seen in the Australian outbreak in the year 2007 when about 76,000 horses were found infected (Cullinane and Newton, 2013). During this outbreak, EIV was also noticed in dogs that were in close proximity to horses but without any lateral transmission (dog-to-dog; Kirkland et al., 2010; Crispe et al., 2011). Similar observation of Transmission of EIV subtype H3N8 to English foxhounds has also been reported during an outbreak in the year 2002 in the United Kingdom (Daly et al., 2008). Most EIV strains isolated recently all over the world originate from the Florida group. Kwasnik et al. (2016) compared available GenBank database of full-length NS sequences of EIV with Florida group. The alignment indicated I194V common in all American lineages and may serve as a discriminator from Eurasian lineages. EIV does not show any seasonal incidences thus can occur at any time of the year (Chambers, 2014; Landolt, 2014). Mortality due to EIV is rare, which can happen in foals devoid of maternally derived antibodies and also in affected horses and donkeys that is devoid of rest. Also, due to the presence of maternally derived antibodies the incidence of this disease is quite low in foals (Landolt, 2014).

Several prototypes of the virus have been isolated in during 1956 to 1989 including influenza A/equine/Prague/1/56 (H7N7), influenza A/equine/Miami/ 1/63 (H3N8), while influenza A/equine/Jilin/1/89 (H3N8), which has emerged by trans-species transmission from birds in China in the year 1989 (Chambers, 1992; Guo et al., 1992). H7N7 was nominated as the prototype EIV and there was no report of isolation of H7N7 after the year 1979 (Webster, 1993). H7N7 has only been reported twice in the Asian continent; first time in Malaysia in the year 1977 and later for the second time in India in the year 1987 based on sero-survey studies (Uppal and Yadav, 1987; Uppal et al., 1987). Disappearance of H7N7 may be explained on the basis of the codon usage concept where it was observed that in this strain possessed strong codon biasness is strong and not guided by mutation pressure or nucleotide composition (Kumar et al., 2016). In the year 1963, H3N8 subtype caused a major epidemic in the USA (Florida), which was later designated as equine subtype 2 (Daly et al., 2004). Evidence suggests that there was a spread of the H3N8 virus from Florida to countries like Australia, Japan as well as China (Murcia et al., 2013; Karamendin et al., 2014). Now, the H3N8 subtype virus mainly circulates throughout the world. South America is considered to be the origin of the spread of H3N8 to other countries (Perglione et al., 2016).

Several reports regarding outbreaks and evolution of the virus are documented throughout the world and comprehensive reports from various such studies from different continents are documented in the following section.

### North america

A/equine/Montana/9564-1/2015 (H3N8) was isolated from an outbreak in USA during 2015 from the unvaccinated equines and sequence analysis showed that the virus was identical to A/equine/Tennessee/29A/2014 (H3N8) based on its polymerase acidic (PA), polymerase basic protein 1 (PB1), hemagglutinin (HA), matrix (M) and nucleoprotein (NP), while analysis of non-structural proteins (NS), neuraminidase (NA), and PB2 showed maximum identity with A/equine/Malaysia/M201/2015 (H3N8). The virus could grow on various primary cells derived from bovine, equine, human, and swine implying that it has the potential to cross species barrier and produces infection (Sreenivasan et al., 2018).

A Canadian study at a racetrack showed 76% prevalence of EIV (Morley et al., 2000). A surveillance study in Ontario, Canada showed that morbidity rate of EIV was 56.6% among the equine respiratory outbreaks. H3N8 was also isolated from 15 horses affected during five different outbreaks (Diaz-Mendez et al., 2010).

A recent sero-survey conducted in West Indies among 140 horses and 40 donkey serum samples revealed 49 samples positive for EI antibodies. This was the first report of EIV infection from Leeward Islands of West Indies (Bolfa et al., 2017).

### South america

Gaíva e Silva et al. (2014) observed seropositivity of EIV in 92% of equines amongst Brazil equine establishments. The high prevalence of antibodies against EIV suggested that the virus circulated extensively among the animals, and statistical analysis indicated that the movement and high aggregation of animals are associated with virus transmission. In the year 2015, EIV outbreak was reported from both the vaccinated and unvaccinated equines in Brazil. Notably, all the 12 isolates recovered during the outbreak were classified as Florida Clade 1 EIV. The reason behind these outbreaks were identified either the use of old vaccine without updation or the use of updated vaccine without proper trail (Favaro et al., 2018).

### Europe

A genetic analysis of HA1 domain of hemagglutinin H3 of EIV isolated during 2005 to 2010 was determined and the genetic evolution of French EIV strains and strains isolated globally was performed (Legrand et al., 2015). The study revealed that EIVs evolved in France during 2005–2010 in a similar manner as other parts of world. Genetic evolution study of all the EIV isolated in France from 1967 to 2015 was studied and it was found that till 2003 American and Eurasian lineages were predominating while the Florida sub-lineage Clade 2 predominated after 2005 (Fougerolle et al., 2017).

Genetic characterization of Italian isolates revealed a close relatedness to American, European, and also the prototype vaccine lineage A/eq/South Africa/4/2003 isolate (Damiani et al., 2008). First incidence of Florida clade 1 virus in Nordic countries was reported in the year 2011 in Sweden which supports the use of both clade 1 and 2 Florida sublineage viruses in the vaccine (Back et al., 2016). HA1 gene of 18 EIV isolated during 2007–2010 in Ireland was carried out and it was found that all isolates belonged to Florida sublineage hence Expert Surveillance Panel recommended the use of both clades of Florida sublineage in the vaccine (Gildea et al., 2012). Later in 2014, EIV outbreaks were reported in 19 premises of Ireland. Though there was clear vaccination history against EIV which may be due to the non-updation of vaccines with Clade 2 of the Florida sublineage (Gildea et al., 2018). Phylogenetic analysis of HA and NA gene of Greek EIV isolates recovered during the year 2003–2007 showed that they are related to Eurasian lineage and Florida sublineage clade 2, respectively. This study suggests that there may be possibility of reassortment (Bountouri et al., 2011). Recently in February 2018 there was report of equine influenza from Scotland and the virus belonged to Florida clade 1 sublineage and this sublineage has not been reported in UK after 2009 (Whitlock et al., 2018).

### Africa

African countries also reported this virus. A study in Nigerian horses showed the presence of H3 and H7 subtypes in their sera by ELISA (Meseko et al., 2016). Later, in a serosurvey conducted in Nigeria employing nucleoprotein-based ELISA, 173 out of 284 animals screened were found to be positive for the presence of EIV antibodies. Thus, a thorough screening is warranted in these areas to carve out the clear picture of the disease status and to adopt better control programs (Meseko et al., 2016). Sequencing of three H3N8 isolates from Morocco namely A/equine/Nador/1/1997, A/equine/Essaouira/2/2004 and A/equine/Essaouira/3/2004 showed that A/equine/Nador/1/1997 had relatedness with European lineage while A/equine/Essaouira/2/2004 and A/equine/Essaouira/3/2004 had homology with A/equine/Fontainbleu/1/1979. A/equine/Essaouira/2/2004 and A/equine/Essaouira/3/2004 also showed 12 substitutions in NS1 protein when compared with the reference A/equine/Miami/1963 strain (Boukharta et al., 2015).

### Asia

During 2007–2008, China and its neighboring countries; Mongolia, India and Japan were invaded by various EIV strains (Qi et al., 2010). Further, phylogenetic analysis revealed that the Chinese strains, Indian strain (Jammu-Katra/6/08) and the Mongolian strain (Mongolia/1/08) were of Florida sublineage clade 2 type. All strains were derived from European strains of this clade as the Newmarket/1/07 and Cheshire/1/07 strains but were unrelated to Japanese strains isolated around the same time (Florida sublineage clade 1) or to Chinese strains isolated in the 1990s (European lineage). There were some unique amino acid changes in the antigenic sites in Asian strains of Florida sublineage clade 2. The loss of a glycosylation site in the Chinese Liaoning/9/08 strain, leads to evolution of few new characteristics (Qi et al., 2010).

Since 2007, several outbreaks of EI have occurred in Kazakhstan, western Mongolia, India, and western China and all these have similarities with EIVs circulating in the same period in neighboring countries (Karamendin et al., 2014). Genetic characterization of the viruses revealed the formation of an EIV cluster and continued evolution of this lineage in central Asia between 2007 and 2012. The main genetic changes observed were in the HA gene without any antigenic drift. Recently, H3N8 A/Equine/Kyonggi/SA1/2011 (KG11) was isolated in Korea which had naturally truncated NS1 protein coding gene (Lee et al., 2017). Recently, genome of two equine influenza strains namely A/equine/Kostanay/9/2012(H3N8) and A/equine/LKZ/9/2012(H3N8) isolated from Kazakhstan was sequenced completely. It was reported that though the isolates were isolated at the same time there was sequence difference at some points indicating the evolution of equine influence (Burashev et al., 2018).

In India, the influenza like symptoms in equines were first reported in 1964 from the Bombay Turf Club, Mumbai, where around 400 horses showed symptoms of coughing (Manjrekar et al., 1965). Since then, India has experienced two major epizootics, first of which was recorded during January to August 1987, which involved over 83,000 equines in north and central India (Uppal and Yadav, 1987; Uppal et al., 1989). Two virus isolates of H3N8 subtype namely Ludhiana/87 and Bhiwani isolate were confirmed during the outbreak in 1987. Second epizootic was reported in 2008–2009 after a gap of 20 years, which initially started from Jammu and Kashmir and covered almost 14 states in the country (Virmani et al., 2008, 2010a,b). Based on the place of isolation, the isolates were named as A/equi-2/Ahmadabad, A/equi-2/Jammu-Katra/08 and A/equi-2/Mysore/08. HA gene of the isolates were analyzed phylogenetically which showed relatedness Florida sublineage Clade 2 in American lineage (H3N8) and also very similar to Chinese isolates of 2007–2008 (Virmani et al., 2010b). Similarly, analysis based on M gene showed homology of 98.41% and 99.54% with other clade 2 Asian origin viruses for M1 and M2 amino acids sequences, respectively. Asian, Chinese and Mongolian isolates had three and four unique amino acid residues in the M1 and M2 proteins (Virmani et al., 2011). Phylogenetic analysis of the NA gene demonstrated that few Indian isolates differed from the Jammu-Katra/06/08 isolate. The Indian isolates were clubbed in Yokohama/10 isolate subgroup together with Chinese, Mongolian, and Kazakhstan isolates (Bera et al., 2013).

In the year 2007, an outbreak had been reported in China among Asian wild horses (*Equus przewalskii*). The virus had been isolated and completely sequenced and then designated as strain A/equine/Xinjiang/4/2007 which showed 99% homology with Florida-2 sublineage rather than with strain A/equine/Qinghai/1/1994 (European lineage) responsible for previous outbreaks in China (Yin et al., 2014). In March 2017, an EIV outbreak in donkeys from Shandong province of China was reported where the virus was found to be A/donkey/Shandong/1/2017 (H3N8) belonging to the Florida sublineage clade 2. Amino acid sequence comparison with the vaccine strain A/equine/Richmond/1/2007 showed substitutions at A, B, and C antigenic regions. The report suggested the circulation of newly emerging EIV in donkeys in China (Yang et al., 2018).

The Japanese EIV isolate Kanazawa/07 phylogenetically relates to American sublineage Florida virus clade (Ito et al., 2008). Some scholars have claimed that the 1889 human pandemic culprit was H3N8 EIV (Xie et al., 2016). An EIV isolate with truncated NS1 gene was isolated in South Korea which belonged to Florida sublineage clade 1. Truncation in the NS1 gene has not affected the replication of the virus (Na et al., 2014).

During the period 2015–2016, an outbreak of EI among equines of several districts of Khyber Pakhtunkhwa Province of Pakistan was noticed. An extensive epidemiological survey was conducted during the outbreak and it was found that A/equine/Pakistan/16 viruses was suggested to be outcome of reassortment between equine and avian influenza viruses as it possessed H3N2 or H7N3 like M and NP genes which was unique compared to other viruses (Khan et al., 2017).

Turkey reported their first EIV outbreak in the year 2013 and the virus was found to be a Florida clade 2 sublineage H3N8 which was similar to the one circulating in Europe (Gahan et al., 2017). Influenza virus A (A/camel/Mongolia/335/2012[H3N8]) has been isolated from camel in Mongolia, which is more evident of expansion in the host spectrum of this virus (Yondon et al., 2014). Survey carried out in the four provinces of Mongolia during 2016–17 showed that seven samples to be positive by qPCR and two samples were suspected positive for EIV out of 680 nasal swabs of horses examined but none yielded growth in MDCK cell line. Similarly, there was no positive case observed out of 131 camels tested. This study reported a sporadic incidence of enzootic EIV in Mongolia (Sack et al., 2017).

### Australia

In Australia, the first outbreak of EIV was reported in the year 2007. The major outbreak appeared in New South Wales and Queensland affecting more than 1,400 equines within a month (Burnell et al., 2014).

### Disease transmission

**Figure 3:**
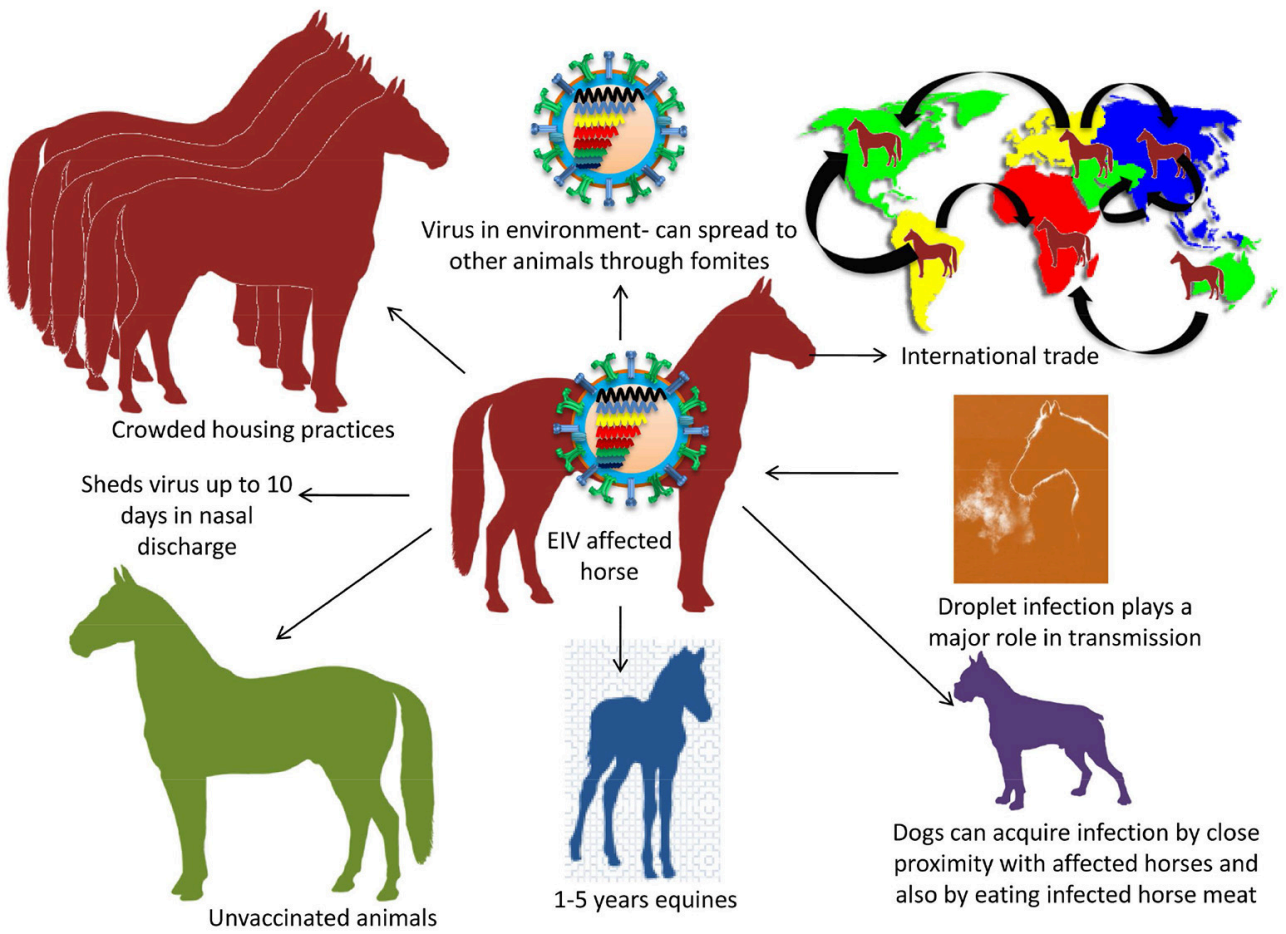
Transmission of EIV. Droplet infection is an important mode of transmission. Transmission between animals includes crowded housing practices, non-vaccination, young horses of 1–5 years and international trade. Dog gets EIV by consuming infected dead horse meat.

The virus transmission occurs by inhalation through aerosol that can spread effectively through air up to 1–2 km of distance. Droplet infection plays a major role in the transmission as nasal discharge/fomites aid in animal to animal transfer (Timoney, 1996; Easterday et al., 1997). Horse-to-horse spread is fairly rapid and faster than other respiratory infections in the equine species (Chambers, 2014). International trade and traffic also leads to spread of disease to disease free zones of the world. Virus can endure for 3 days in the environment leading to the spread in other animals through fomites. The incubation period is 1–3 days and the infected horses have been found to shed the virus up to 10 days via nasal discharge (Daly et al., 2004). Crowded housing practices of equines usually aid in the fast spread of the EIV (Figure 3).

Being a contagious disease, the rate of EIV infection is almost 100 per cent in unvaccinated horses. Age groups of 1–5 year-old naïve equines are more susceptible to EIV. Immune status of the animals plays an important in the onset of disease. Partially immune animals tend to become infected sub-clinically. Further, the spread of virus in partially immune animals is slower than in naïve animals (Landolt, 2014). EIV is a self-limiting sterile disease in horses since the virus does not persist in recovered animals (Cullinane and Newton, 2013). It was speculated that interspecies jump to dogs might be due to proximity with infected horses as happened in the first report of H3N8 in dogs in Florida, 2004 and/ or by eating the infected horse-meat (Crawford et al., 2005; Newton et al., 2007).

### Cross-species transmission

Influenza virus shows partial host restriction and this characteristic is attributed to the HA gene. HA as a viral receptor binding protein, binds to the sialic acid (SA) host cell receptors. Binding is dependent upon the SA species (N-acetylneuraminic acid or N-glycolneuraminic acid) and its linkage with galactose moiety; either α(2 → 6) linkage or α(2 → 3) linkage. Human influenza viruses prefers SA α(2 → 6)-gal in N-acetylneuraminic acid form, whereas avian, equine, and canine influenza viruses prefer binding to SA α(2 → 3)-gal in N-glycolylneuraminic acid form (Ito and Kawaoka, 2000). HA gene analysis has thrown much light on the evolution of equine and canine influenza viruses (Shi et al., 2010). Comparing the sequences of EIV isolated from equine and canine host showed differences or changes that occurred to meet the requirement for host cell adaptation and tropism. But not much change has been found to occur in the influenza virus biology during virus interspecies jumping (Collins et al., 2014; Feng et al., 2015). The canine influenza virus (CIV), A/canine/Colorado/30604/2006 (CO06, H3N8) evolved from an equine strain found to be only mildly pathogenic in equines due to a change in receptor preference acquired for viral entry into the host cell during mutation (Yamanaka et al., 2010). EIV have limited host specificity with the exception of reports of H3N8 virus transmission in dogs (Kirkland et al., 2010). A limited transmission of EIV H3N8 has been reported among dogs in the United Kingdom as well as Australia (Parrish et al., 2015). The equine and canine H3N8 viruses have minimum difference biologically and both show mutation in PA-X protein (Feng et al., 2015, 2016). Simultaneous circulation of H3N8 viruses in dogs and horses makes bi-directional virus transmission possible (Rivailler et al., 2010). Studies have indicated the inability of H3N8 CIV isolates to replicate and spread in equids and suggested the involvement of factors other than receptor binding specificity in infection with EIV (Kirkland et al., 2010; Pecoraro et al., 2013; Landolt, 2014). Reciprocally, equine H3N8 lineages are absent in dogs (Rivailler et al., 2010). It is interesting to note that due to substitution of amino acid in the binding site there may be modification in the H3N8 replication in the canine respiratory tract (Collins et al., 2014). In line, experimental transmission of EIV H3N8 to cats has also been reported (Su et al., 2014), indicating the wider host spectrum of EIVs in the event of exposure. In the year 2012–2013, EIV (H3N8) was also isolated from a Bactrian camel in Mongolia (Yondon et al., 2014). Out of 460 nasal swabs only one isolate A/camel/Mongolia/335/2012[H3N8] was recovered highlighting the need for further investigations in camels to find the pathobiology of EIV in camels.

The phylogenetic analysis of H3N8 viruses isolated from dogs and horses revealed monophyletic and distinct evolution of both viruses. However, analysis of a limited number of EIVs suggested substantial separation in the transmission of viruses causing clinically apparent influenza in dogs and horses (Rivailler et al., 2010). Yamanaka et al. (2009) tested the possibilities of interspecies transmission of EIV to dogs due to close contact with experimentally EIV infected horses. The infected horses were kept with healthy dogs in three groups in close proximity for 15 days and HI test revealed sero-conversion in all with viral shedding in dogs of two groups without apparent clinical symptoms. The same study was performed in inverse order by Yamanaka et al. (2012a) with healthy horses and CIV infected dogs kept in close contact to investigate the interspecies transmission. Though all the dogs infected with CIV presented clinical signs of lung consolidations after euthanasia, none of the horse showed clinical signs, virus shedding, seroconversion or lesions in the respiratory tract. These findings thus revealed that a single dog infected with CIV is not sufficient enough to be a source of CIV infection in horses. Short et al. (2015) have reviewed influenza A viruses comprehensively by describing interspecies virus transmission and analyzing the current knowledge regarding adaption of influenza viruses to a new host.

Two isolates of H3N8 EIV were isolated from swine in China when pigs were screened for swine influenza (Tu et al., 2009). Though pigs possess both α-2,6 galactose and α-2,3 galactose sialic acid receptors, a study with EIV does not produce fever and other notable histopathological changes. H3 HA of influenza viruses has extended pathogenic potential, but analysis showed that evolution of H3 from equine and canine origin is different from H3 of swine, avian, and human viruses (Shi et al., 2010). Further, the potential of H3N8 influenza virus from canine, equine, avian, and seal origin has been tested for its capability to infect pigs. Avian and seal H3N8 viruses replicate substantially causing detectable lesions in pigs without previous adaptation. No specific antibodies against hemagglutinin in any H3N8 infected pigs could be detected. Therefore, special attention is required toward viruses of the H3N8 subtype since these may infect pigs without detectable anti-hemagglutinin antibodies and thereby pose a risk of genetic reassortment where pigs act as mixing vessel for influenza viruses (Solórzano et al., 2015).

### Equine influenza and human infection

Although there are sparse reports of EIV infection in man, the data regarding the phenomenon originated from Mongolia, where horse-to-man population ratio is highest in the world. It is presumed that the probable cause of human pandemic in year 1889 is supposed to be due to the involvement of H3N8 EIV (Elbadry et al., 2014; McAuley et al., 2015). However, to note due to close association (both temporal as well as geographical) between human as well as equines, influenza-like disease epizootics had been observed before the advent of various serological and molecular assays to detect the virus (Morens and Taubenberger, 2010). It is reported that the development of influenza-like illness (ILI) among Mongolian children occurred after exposure to infected equines (Khurelbaatar et al., 2014). Few anecdotal reports are also suggesting the suffering of Mongolian children with ILI due to the exposure to EIV infected horses (Xie et al., 2016). After the H3N8 epizootic in New South Wales and Queensland, Australia in 2007, a cross sectional study was carried out enrolling 89 humans exposed to infected horses along with 11 controls. Hemagglutination, micro-neutralization, and enzyme-linked lectin assays were carried out in serum samples to detect H3N8 antibodies, but with a low titer of antibodies indicating the absence of acute infection, which could be the outcome of cross reacting antibodies (Larson et al., 2015). Also, the experimental data revealed that the H3N8 virus could not attenuate following passage in humans, as it was still capable of infecting and causing illness in horses (Couch et al., 1969). In a cohort study conducted among Mongolian adults, after occupational exposure to EIV infected horse, ILI was observed, and upon quantitative real time PCR and virus culture, 36% ILI cases were found influenza A positive without evidence of EIV (Khurelbaatar et al., 2013, 2014). During Australian outbreak of EI in the year 2007, human samples collected showed only little seropositivity for the equine strain, which was concluded by the fact that human vaccines had cross-reactivity to this virus or humans were not susceptible to EIV (Burnell et al., 2014).

## Equine influenza: the disease

### Clinical manifestations

Clinical signs of EI include the loss of appetite, fever, general weakness, poor performance, harsh dry cough, hyperemia of nasal and conjunctival mucosae, tachycardia, dyspnea, stiffness in legs due to limb edema and muscle soreness, enlarged lymph nodes, serous nasal discharge, which may turn yellowish due to secondary bacterial infection and abortion. There is high morbidity rate in EI while mortality rate is low, and death usually occurs due to pneumonia as a sequela. In rare cases, myocarditis and chronic obstructive pulmonary disease is seen especially when horses return to training too soon. Encephalitis in horses and rapid fatal pneumonia in foals and donkeys has also been recorded but its pathogenesis is not clear (Gerber, 1970; Radostits et al., 2003; Newton and Mumford, 2005; Cullinane et al., 2006; Daly et al., 2006; Landolt et al., 2007). Both the subtypes produce similar clinical symptoms, but these are more severe in case of H3N8 infection (Chambers, 2014). Incubation period usually depends on the immune status of the animals (varies from 18 h to 5 days under experimental settings) and can be very short, up to 24 h in naïve horses (Cullinane and Newton, 2013). Fever of 39.4–41.1°C may last for 2–3 days. Carrier status does not exist, but sub-clinical infection in recently vaccinated animals may go unnoticed. Usually horses recover within 1–2 weeks, while severely sick animals require a month time to recover (Cullinane and Newton, 2013). Clinical signs in dog include fever, cough, occasionally suppurative bronchopneumonia and per acute death (Crawford et al., 2005).

### Pathogenesis and pathology

**Figure 4:**
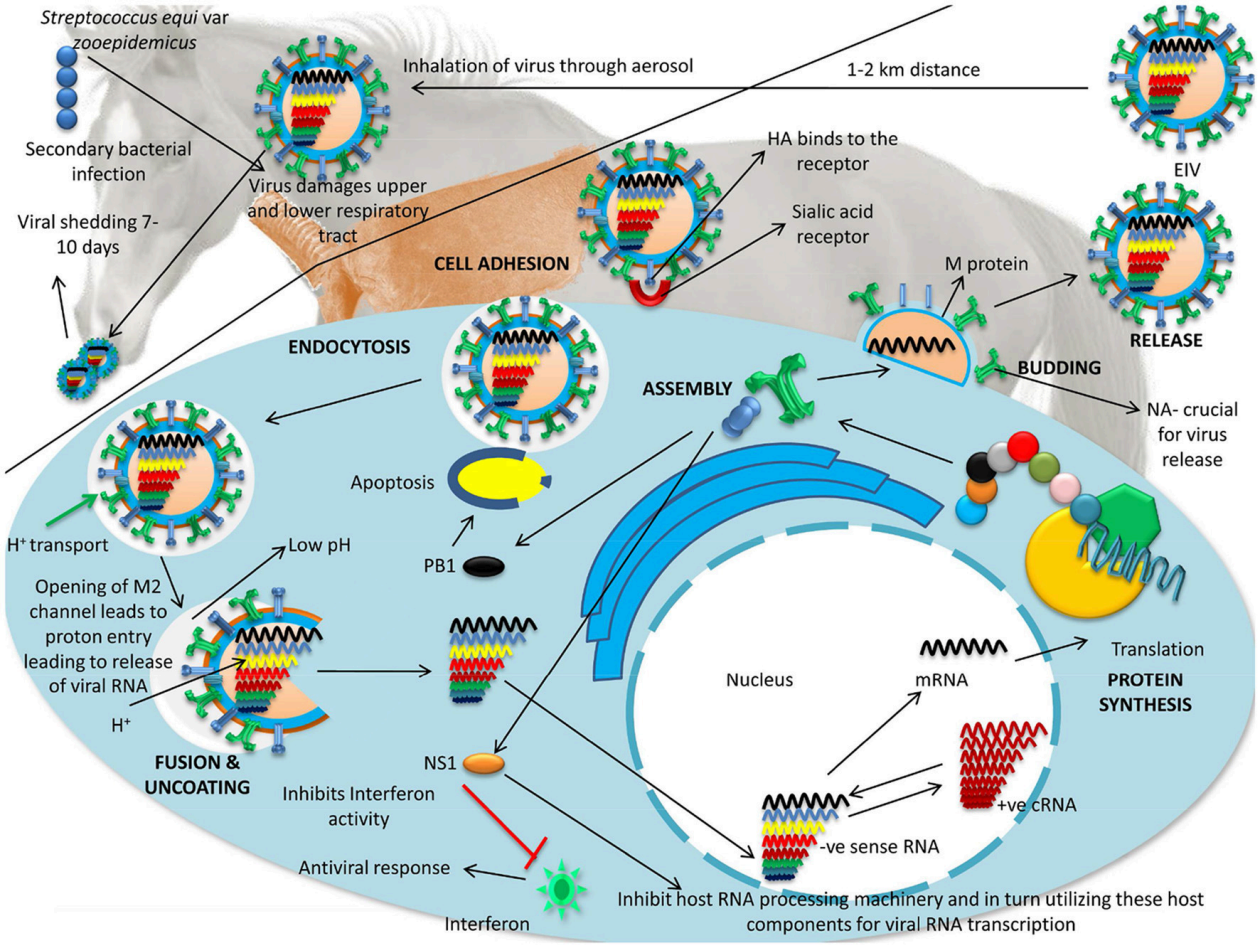
Replication and pathogenesis of EIV. EIV damages the upper and lower respiratory tract's ciliated epithelial cells thereby causes inability to clear foreign substances. Spike glycoprotein HA fastens to the receptors present on the respiratory epithelial cells and it enters the cells by endocytosis. After endocytosis, EIV undergoes fusion and uncoating. Opening of M2 channel leads to proton entry and subsequent release of viral RNA followed by synthesis of viral structures leading to assembly of EIV. EIV is released from the infected cells by the process of budding.

EIV mainly damages the upper and lower respiratory tract's ciliated epithelial cells thereby causing inability to clear foreign substances. The spike glycoprotein, HA, attaches with sialic acid receptors localized on host cell surface and subsequent receptor-mediated endocytosis process proceeds to deliver the virus particle inside of cell and remain in an endosome. Lower pH environment in this cell apartment triggers fusion process of membranes of the virus and endosome. Acidic pH alters not only alters the conformation in HA0 but also opens the M2 ion channel and acidifies the viral core and the vRNP (composed of proteins NP, PA, PB1, and PB2) enters the nucleus through host cell's cytoplasm. The viral RNA dependent RNA polymerase (RdRp) initiates the RNA synthesis internally on viral RNA and utilizes host cell's machinery for its own purpose. After completing the viral proteins synthesis vRNPs leave the nucleus all that is left for the virus to do is form viral particles and leave the cell. Being an enveloped entity, it comes out the host cell's through budding (Radostits et al., 2003; Figure 4). Replication of EIV leads to virus particles being released from one cell to enter another cell in the airway, thereby damaging the respiratory tract leading to necrosis of the respiratory epithelial cells, protein rich fluid exudation into the airways, cilia getting clumped and impairing the muco-ciliary apparatus.

Horse respiratory epithelium possess high level of Neu5Gc2-3Gal moiety, which is essential for replication of the virus (Suzuki, 2000). EIV NA possess higher affinity for this moiety. This is important at the early stage of infection in order to release the progeny from the infected cell (Takahashi et al., 2016). Cells infected with EIV undergo apoptosis, which is the common pattern of cell death (Hinshaw et al., 1994). Activation and cleavage of caspase is essential for cytotoxicity caused by the virus (Lin et al., 2002). Non-structural protein 1 (NS1) of EIV is pivotal in disease pathogenesis and differences observed in disease severity due to variation in this protein (Elton and Bryant, 2011). NS1 facilitates virus replication along with having an inhibitory effect on anti-viral strategies applied by the host. Viral replication is supported by NS1 by inhibiting host RNA processing machinery and in turn utilizing these host components for viral RNA transcription preferably. NS1 further inhibits the host anti-viral response by preventing activation of various host defense components such as interferon regulatory factor 3 (IRF-3), NF-kB, and other transcription factors (Barba and Daly, 2016). Cytotoxic T cell response is generated against M, NP, and PB2 which helps in viral clearance (Landolt, 2014). Of interest, eukaryotic cells express a wide range of small regulatory RNAs, including miRNAs that have the potential to inhibit the expression of mRNAs (e.g., viral) showing sequence complementarity. However, a study hypothesized that human viruses including influenza A viruses might have evolved in a manner so that it resists endogenous inhibition by miRNAs (Bogerd et al., 2014).

Viral shedding can be observed for a period of 7–10 days, while viral genome (RNA) can be detected for 15 days or more by PCR (Chambers, 2014). This infection is seldom fatal in horses except in colostrum (specific immunoglobulin rich) deprived newborn foals (Chambers, 2014). Due to accumulation of fluid in the respiratory tract, there are chances of secondary bacterial infection and organisms like *Streptococcus equi var. zooepidemicus* increases the inflammation leading to bronchopneumonia (Radostits et al., 2003). Thus, EI disease is further worsened by concurrent bacterial infection acting as a helping hand in fatal episode of equine influenza (Daly et al., 2004). Respiratory epithelium takes around 3 weeks to recover which aggravates or provide gateway for secondary bacterial infection (Landolt, 2014; Na et al., 2016).

Bronchiolitis is the common lesion noticed with serous exudates bilaterally. Gross pathology reveals diffuse to very extensive pulmonary consolidation and histological changes like necrosis of bronchioli and alveoli, infiltration of neutrophils, formation of hyaline membranes and airway epithelium undergoing hyperplasia and squamous metaplasia (Patterson-Kane et al., 2008). Studies revealed that a secretory layer of mucous is present in the nasal cavity of the equine species that can prevent the attachment of HA protein of influenza virus. Similarly, the nasal passage also possesses sialo-receptors that can mask receptors specific to influenza (Scocco and Pedini, 2008). EIVA/equine/South Africa/2003 in dogs revealed similar pathology as that of CIV causing extensive damage to the respiratory epithelium. This damage was more extensive than that caused by 1963 EIV in canines. This shows that the recent EIV strains can infect dogs readily (Gonzalez et al., 2014). Pavulraj et al. (2015) have studied the pathology of EIV (H3N8) in a murine model. The pattern of disease progression, lesions and virus recovery from nasal washings and lungs in mice were found comparable to natural and experimental EIV infection in equines. These findings establish BALB/c mice as an attractive small animal model for studying EIV (H3N8) infection. Authors also reported that BALB/c mice could be a better candidate for testing the EIV vaccines before trial in equines (Pavulraj et al., 2017).

### Diagnosis

There is an array of diagnostic techniques available for EIV infection *viz*. isolation of virus, serological assays, antigen and genomic RNA detection (Mumford, 1990; Chambers et al., 1994; Gupta et al., 2003; Cullinane et al., 2006; Landolt et al., 2007; OIE, 2008; Cullinane and Newton, 2013; Kapoor and Dhama, 2014). However, the clinical diagnosis of EIV remains a challenge, where high fever and dry cough are predominantly noticed. The differential diagnosis includes other respiratory diseases like equine adenoviral infection, equine herpesvirus infection, equine rhinopneumonitis, equine viral arteritis, travel sickness (pleuropnemonia), and also strangles (Radostits et al., 2003).

Swab samples from nasopharyngeal region can be taken immediately after the onset of clinical symptoms (i.e., within 3–5 days). Choice of sampling must be very careful as it affects the accuracy of results. Nasopharyngeal swab yields more density of virus than the nasal swabs (Chambers and Reedy, 2014a). Transportation of the samples in appropriate ice-cold containers is essential. If samples are to be transported for more than 2 days, these should be kept at −60°C or at lower temperatures. Allantoic or amniotic routes are performed for isolation of EIV and fluids from these cavities are subjected to HA and a titer of more than 16 is considered positive. If the titer is low then further blind passages have to be given. Isolation of virus is usually done in embryonated chicken eggs by amniotic route. Chorioallantoic membranes can also be used for isolation of the virus and instead of HA, qRT-PCR can be employed for detection of the virus rapidly (Gora et al., 2017). Cell lines like Madin Darby canine kidney (MDCK) are suitably used for virus isolation (Easterday et al., 1997). Both chicken egg and MDCK cell lines allow viral mutations, but embryonated eggs are generally preferred for isolation due to comparatively lesser heterogeneity in them (Chambers and Reedy, 2014b). Samples that show isolation negative results should be passaged again and up to five blind passages may be necessary for samples from vaccinated horses.

Seroconversion can also be employed for diagnosis of the disease and assays such as HI, single radial hemolysis (SRH), single radial immunodiffusion (SRID) and enzyme linked immunosorbent assay (ELISA) are commonly used (OIE, 2008). Antigen capture ELISA for H3N8 virus using a monoclonal antibody against nucleoprotein can be employed at places lacking facilities for isolation of the virus (Cook et al., 1988; Livesay et al., 1993). ELISA to detect antibodies against nucleoproteins can be used to differentiate infected animals from vaccinated ones as the same did not detect antibodies generated after canary pox vectored vaccines for this virus that contain HA protein (Kirkland and Delbridge, 2011; Galvin et al., 2013). It has been suggested to use ELISA (cell-based) for measuring anti-non-structural (NS1) protein antibodies which has found its application in differentiating infected from vaccinated animals (DIVA) in equines (Rozek et al., 2011). Characterization of the isolate can be done by HI using specific antisera, but due to the presence of inhibitors of hemagglutination (such as α-2 macroglobulin) in equine sera complicates the interpretation of this test (Chambers, 2014). This can be overcome by pre-treating the sera with Tween-80 and ether or Kaolin (Chambers, 2014; Chambers and Reedy, 2014c). For identification of susceptible population of horses and for the purpose of disease investigations related to outbreak in immunized horses, single radial hemolysis (SRH) test has been found to be useful (Gildea et al., 2010, 2011). SRH tests give higher correlation between serum antibody titer and protection level from the disease and also indicate sterile immunity (Chambers, 2014; Chambers and Reedy, 2014c). SRID is a higher version of chick cell agglutination test and there is no high variation among tests (Wood et al., 1983). Paired serum samples should be used for serological tests and seroconversion (as reflected by a four times higher antibody titer) indicates recent influenza virus infection.

Tests aiming at genomic material detection like reverse transcription-polymerase chain reaction (RT-PCR) and real-time PCR can be used for EIV diagnosis (Donofrio et al., 1994; Foord et al., 2009; Read et al., 2012). A qRT-PCR test for the matrix gene of type A influenza viruses was used during the 2007 Australian EI outbreak to confirm the presence of the virus in animals to be exported (Diallo et al., 2011). However, at the end of the outbreak, four horses were found positive due to contamination of the samples with the vaccine (Diallo et al., 2011). A real-time RT-PCR (rRT-PCR) assay has also been employed for the detection of virus in the nasal swab of horses in Mongolia (Yondon et al., 2013).

The use specific primers in RT-PCR assay for conserved region of this virus was found to give rapid results with high sensitivity even in case where viral load in excretions was very less. Furthermore, viruses of unknown lineages could also be detected by using such primers (Aeschbacher et al., 2015). Multiplex RT-PCR test to detect H3N8 has been developed which is expected to detect newly EIVs (Lee et al., 2016). In one study, it was found that DFA (rapid antigen detection test kit) used to detect nucleoprotein in nasal swabs is a very sensitive antigen test and can act as supportive test for laboratory diagnosis of EIV in clinical samples (Galvin et al., 2014). Using specific primers, EIV typing can also be done and RNA-DNA hybridization test has also been reported (Gupta et al., 2003). Nested-PCR has also been developed which showed that it is useful for diagnosis of EIV (Oxburgh and Hagström, 1999). Immuno-PCR, a more sensitive assay than the RT-PCR has been developed for detection of NS1 protein (Ozaki et al., 2000). Real-time light cycler RT-PCR has been reported to be more sensitive than either the isolation of virus or ELISA (Quinlivan et al., 2005).

RT-PCR followed by sequencing has been used for diagnosis and subtyping of the neuraminidase (Alvarez et al., 2008). TaqMan RT-PCR targeting nucleoprotein (NP), matrix (M) and HA gene of both H7N7 and H8N8 subtypes has also been developed. The results of this developed assay do not cross react with any of the other known equine respiratory viruses (Lu et al., 2009). Reverse transcriptase-loop mediated isothermal amplification (RT-LAMP) has been developed to detect HA gene of both H3N8 and H7N7 EIV, which is more sensitive than RT-PCR. Further, this test can also be used to differentiate H3N8 and H7N7 in clinical samples (Nemoto et al., 2011, 2012). Recently, insulated isothermal RT-PCR (iiRT-PCR) has been developed to detect HA3 gene of EIV, which is a highly sensitive and specific test (Balasuriya et al., 2014). The test involves three steps viz. rehydration of lyophilized reagents, sample nucleic acid addition and then placing on POCKIT™ Nucleic Acid Analyzer device. This test is rapid as it requires only 1 h to complete the reaction on this portable device and does not require any post-amplification processing (Balasuriya, 2014; Balasuriya et al., 2014). Pyrosequencing has also been recently implicated in identifying clade differentiation of EIV at the time of outbreak (Bernardino et al., 2016). Many rapid antigen detection tests (ImmunoAce Flu, BD Flu examan, Quick chaser Flu A, B, ESPLINE Influenza A&B-N, etc.) have been developed for diagnosis of this virus. However, the sensitivity of these tests is very low, also they detect virus for short period of time as compared to the molecular tests such as RT-PCR thus giving higher rate of false negative results (Yamanaka et al., 2008b, 2016a). A sensitive silver amplification immunochromatography was developed for early detection of EIV (Yamanaka et al., 2017a).

## Prevention and control

### Vaccines

To deal with emerging viral diseases of equines including EI, it is mandatory to strengthen the medical/veterinary services with adopting appropriate preventive measures such as vaccines and adjuvants (Bayry, 2013; Chambers and Balasuriya, 2016; Paillot et al., 2017). It has been seen that vaccination has been practiced since 1960s, however, its efficacy is still a matter of debate due to the use of less potent vaccines, improper vaccination schedule and also use of outdated virus strains, and due to continues drift in the viral genome (Bryans et al., 1966; Minke et al., 2004; Mathew et al., 2006; Meeusen et al., 2007). Hence, use of the virus from the most recent outbreak as vaccine renders better protection (Barbic et al., 2009). Moreover, experience from the field cases has shown that vaccination was successful in preventing H7N7 infection but outbreaks due to H3N8 are prevalent both in vaccinated and non-vaccinated animals. H3N8 subtypes are the main cause of EIV infection and vaccine virus different from prevailing subtypes leads to subclinical infection, which is followed by viral shedding from vaccinated animals as well. This further contributes to the spread of disease (Daly et al., 2004). Influenza A viruses are able to evade host immunity even in vaccinated horses, and study of intra- and inter-host evolution of EIV in vaccinated horses, revealed the similar level and structure of genetic diversity with those in naïve horses. However, intra-host bottlenecks were more stringent in vaccinated animals and mutations were present near putative antigenic sites as shown by Murcia et al. (2013). The virus isolates collected from the outbreak area determines the vaccine strain selection, and hence essentially surveillance programmes should have sufficient funding with active involvement of equine veterinarians (Elton and Cullinane, 2013).

Antigenic drift at the HA gene (a major protein of influenza A virus) led to vaccine failure in various parts of the world (Mumford and Wood, 1993; Wilson, 1993). Genetic reassortment taking place during a mixed infection can lead to the development of new strains and ultimately vaccine failure (Bryant et al., 2009). To deal with this problem, continuous checks and monitoring through surveillance programs and updating of vaccines with recent strains remains the best and effective way in prevention and control of this disease. Such methods have proven to be successful in Ireland during 2007–2010, where regular surveillance provided scientific or effective control of EI disease. The study revealed that the EIV strain changed from prevalence of Eurasian lineage to clade 1 and clade 2 of Florida lineage. Accordingly, changes made in vaccine strains lead to scientific or effective control of the disease (Gildea et al., 2012). Similar results were obtained by proper surveillance programs in South America where incidence of this disease have reduced prominently after incorporation of Florida clade 2 strains in vaccines (Perglione et al., 2016). A study was conducted in United Kingdom to know the antigenic changes occurred in EIV isolates during 2013–2015 so as to get a clear picture on the efficacy of vaccine. Result showed that Florida sublineage clade 2 was diverging. The study also suggested inclusion of Florida sublineage clade 1 and 2 in the vaccine for EIV (Rash et al., 2017). Thus, an epidemiological surveillance of influenza virus along with monitoring of impact of immunization is extremely important. The disease control is influenced by antigenic variation of the virus, target group, goal of immunization, rate of antigenic variation, and vaccine composition (Horspool and King, 2013).

There is protection of ponies from vaccinated dams but antibodies titer decline as the days passes, hence it is essential to protect the ponies by vaccinating with the recent virus strain prevalent or circulating in the population (Townsend et al., 1999; Meeusen et al., 2007). Vaccination will not completely eliminate the chance of infection and booster vaccines are essential to keep the disease under control (Nelson et al., 1998; Daly et al., 2004; Yamanaka et al., 2008a). Herds with 75% vaccination coverage exhibit better disease control exposed to virulent infections. Considering vaccine efficacy, it is important to assess it's HA content because it is the main component that determine viral entry into the cell (Daly et al., 2004).

The World Organization for Animal Health (OIE) is the apex body to decide the strain to be used for vaccination in commercial vaccine preparations. Every year the molecular data of HA gene sequencing is taken into account and antigenic characterization is carried out by using reference sera to take cross protection studies results into account. This data is reviewed by Expert Surveillance Panel (ESP) constituting members from WHO and OIE. Then, they suggest whether there is a need to update the existing vaccine. Such program however failed by the disparity in the level of surveillance carried out in different countries (Cullinane et al., 2010). New strains are included only in case, where previously recommended strains are not providing optimum protection. Also, clinicians must be vigilant enough to decide which vaccine strain to be incorporated in the prophylactic regimen (Cullinane et al., 2010). Regular monitoring is required so that any mutation in the circulating virus needs to be identified timely and thereby the available vaccines can be updated accordingly. Recently in the year 2016, EIV vaccine in Japan has been updated with A/equine/Yokohama/aq13/2010 and study showed that combining both old and new vaccine provide better protection (Yamanaka et al., 2017b). Complete genomic sequences of both the vaccine strains namely A/equine/Yokohama/aq13/2010 and A/equine/Ibaraki/1/2007 that is being used in the vaccine strain in Japan from 2016 has been published very recently (Nemoto et al., 2018). Vaccines must be administered strategically and appropriately to obtain optimum immune response and desired protection against EIV in equines (Daly and Murcia, 2018).

To update the existing inactivated EI vaccine used in Japan it was decided to include Florida sublineage clade 2 virus. Study was conducted employing A/equine/Carlow/2011 (H3N8), A/equine/Richmond/1/2007 (H3N8) and A/equine/Yokohama/aq13/2010 (H3N8) and the results showed that A/equine/Yokohama/aq13/2010 had higher HI titer hence it was considered to be the better strain for updating the existing vaccine (Gamoh and Nakamura, 2017). If other countries also conduct such studies similarly then it will be very useful for them to implement such strategy when necessary to save equine population from the havocs of an outbreak.

The below section details the advances in designing and developing EIV vaccines and vaccination strategies that are actively being used for equine immunization.

#### Killed/inactivated vaccine adjuvanted with ISCOM-matrix (Prequenza®)

Commercially available EIV vaccines are killed vaccines of whole cell H7N7 and H3N8 subtypes (Park et al., 2003). The inactivated vaccines protect horses from disease with no viral shedding. Inactivated vaccines require booster regimen for better efficacy and are best suited for vaccinating dams so as to protect foals from infection. Formaldehyde, β-propriolactone, ethylene-imine, and thimerosal are frequently used for inactivating viruses for vaccine formulations. An inactivated EI vaccine elicits protective response against circulating strain of Florida clade 2 sublineage. Duvaxyn IE-T Plus® inactivated virus vaccine was given at the interval of 4 weeks and protection from clinical symptoms was observed with reduced viral shedding (Paillot et al., 2013a). Starting at 6 months of age, the vaccine is re-administered at an interval of 3–12 months based on the risk of infection. Killed vaccine adjuvanted with ISCOM-matrix (Prequenza®) has been formulated and found to provide longer duration of immunity (Bengtsson, 2013). This vaccine was found safe to use in pregnant mares and foals (Heldens et al., 2009). Equip™ F (Schering Plough Animal Health, Hertfordshire, UK) vaccine contains a H7N7 strain and two H3N8 strains, each from American lineage as well as Eurasian lineage. It is an ISCOM-based vaccine which significantly reduces the clinical symptoms and prevent virus shedding in ponies (Paillot et al., 2008). It induced EIV-specific IFN-γ production through activating Th1 cells (Paillot et al., 2006a). The study of Paillot et al. (2010) was focused on immunity in absence of updated vaccine strain. Ponies were immunized twice with commercial vaccine Duvaxyn IE-T Plus® with an interval of 14 days and challenged in a containment facility by exposure to a nebulized aerosol of a genetically different strain. The results indicated reduction in pyrexia and virus shedding. However, duration of protective immunity is shorter for a non-updated vaccine and is prone for any further antigenic drift.

Adjuvants in vaccine are immune stimulating components, which aid in boosting humoral and cell mediated response (Horspool and King, 2013). Aluminum salts like aluminum phosphate and aluminum hydroxide, organic adjuvants like squalene, oil-based adjuvants like MF59, mycobacterial adjuvant like Freund's complete adjuvant and monophosphoryl-lipid A/trehalose dicorynomycolate (Ribi's adjuvant) are few good examples of adjuvants. They are aimed at presenting antigen to immune cells, targeting toward antigen presenting cells, and enhancement of cell-mediated immunity (Edelman, 2000). Now-a-days, it is suggested to use A/equi-1 and A/equi-2 strains similar to American and European lineages (Heldens et al., 2004). Certain vaccine virus strains used in inactivated vaccines show cross-protection as observed in case of Japanese strain A/equine/La Plata/1993 (LP93) and Florida lineage strains [A/equine/Carlow/2011 (CL11)] (Yamanaka et al., 2016b). However, Japanese strain A/equine/La Plata/1993 used in vaccine was not able to generate cross-neutralizing antibodies against Florida sublineage clade 2 (isolated from Ireland and UK) EIV due to single substitution (from alanine to valine) at 144 position in the antigenic A site of the HA gene (Yamanaka et al., 2015). Administration of combined inactivated equine influenza virus vaccine with equine herpes virus vaccine has shown increased immune response against EIV (Gildea et al., 2016). Few associated disadvantages with these inactivated vaccines are their poor immunogenicity and predominant short-term humoral immunity, which necessitates repeated immunization (Heldens et al., 2004).

In India, development of a low cost indigenously developed EIV vaccine was necessitated due to the suffering of more than 83,000 equines during 1987 (Uppal and Yadav, 1987). Indian Council of Agricultural Research-National Research Centre on Equines (NRCE) in collaboration with Animal Health Trust, Newmarket, U.K. has developed an EI vaccine employing A/eq/Ludhiana/87 isolate. Vaccine induces satisfactory humoral and protective immunity when challenged with live A/ Equi-2 viral isolates (Sussex/89 and Ludhiana/87) when administered in two doses 4 weeks apart. To prepare inactivated EIV vaccine, isolates from various regions of the country *viz*. Ahmadabad (Gujarat), Katra (Jammu), Gopeshwar (Uttarakhand), and Mysore (Karnataka) were cloned and A/eq/Katra (Jammu)/06/08 (H3N8) virus was used for updating the vaccine on the basis of sequence analysis. Inactivated vaccine along with aluminum hydroxide gel adjuvant was found to be protective and safe as tested in guinea pigs and horses. Vaccine trials conducted in 150 field horses showed development of protective immunity, without any untoward signs. Subsequent trials in thoroughbred horses also gave encouraging results as all the six animals showed protective immune response after booster dose (NRCE Annual Report, 2010).

#### Subunit vaccines

Subunit vaccines encompass purified viral antigens. Among these, two main vaccines are the immune-stimulating complexes (ISCOM)-based vaccines or ISCOMATRIX vaccines. ISCOM based vaccines have ISCOM particles with cage like structures formed spontaneously by viral protein combination with cholesterol, phospholipids and Quillaja saponins. ISCOMATRIX are essentially like vaccines but don't possess cage like structure (Elton and Bryant, 2011). ISCOM based EIV vaccine induces strong antibody response along with elevated levels of IFN-γ (Paillot et al., 2008). Use of ISCOM based EIV vaccine through intranasal route in systemic prime/mucosal boost vaccination program gave transient higher virus-specific IgA in nasal wash (Crouch et al., 2005). Since inactivated vaccines against EIV produce short-term antibody response other methods like ISCOM based vaccine technology has been developed in order to simulate natural infection. Cell mediated immune response is also stimulated by ISCOM based vaccines for EIV as indicated by high IFN-γ production in peripheral blood lymphocytic cells (Paillot and Prowse, 2012). A study using A/eq/Kentucky/98 ISCOM based vaccine administered intramuscularly gave good protection when challenged with reference virus H3N8 of American lineage (Crouch et al., 2004). Recently, a subunit vaccine and a DNA vaccine based on HA stem region of A/equine/Argentina/1/93 (H3N8) virus was tested in mice and it showed 100% protection when challenged with equine strains while 70–100% protection with human strains (H3N8). The study also showed that challenge with human strain H1N1 (A/PR/8/34) did not protect the vaccinated animals, hence the developed vaccine protects animals only against homosubtypic strains (Ibañez et al., 2018).

#### Cold adapted (Ca) vaccines

These vaccines have been developed to aim at improving both humoral and cellular immunity, therefore mimicking the protective immunity generated by natural infection (Paillot et al., 2006b; Paillot, 2014). The Ca EIV vaccine strain is able to replicate efficiently in upper respiratory tract to generate local and systemic immune responses. The most advantageous part is that the Ca strain doesn't replicate in the lower respiratory tract, the niche of wild type influenza virus and therefore symptoms like bronchitis, pneumonia, and pulmonary edema do not occur (Townsend et al., 2001).

#### Modified-live cold-adapted equine influenza A2

These vaccines are administered intra-nasally and have been found to be safe and reduced the onset of EIV outbreak (Chambers et al., 2001; Townsend et al., 2001). Intranasal vaccine resulted in local protection against EIV though the circulating level of antibody reduces as time progresses. Though, administration of this vaccine to yearlings was found to be safe, they are not recommended for use in pregnant mares in late gestation. *In vitro* studies demonstrated that this vaccine could generate cell-mediated immune response in yearlings and pregnant mares 14 days post-vaccination (Tabynov et al., 2014).

#### Canarypox vector vaccines

These vaccines are available to be administered intra-nasally, while boosters are needed at an interval of 6 months (Minke et al., 2007). These vectored vaccines produce a good amount of colostral antibodies; hence are suitable for vaccination of mares at late gestation (Daly et al., 2004). The canarypox-vectored vaccine (ProteqFlu®, Merial) was chosen to vaccinate horses in the United States of America because it can evoke antibodies against HA only. As a consequence, in diagnostic ELISA, NP can be detected and discrimination between infected and vaccinated animals is possible (Daly et al., 2011; Paillot and El-Hage, 2016). Also, such vaccines can induce an immune response early and for longer duration of time against American lineage of EIV (Soboll et al., 2010). Since canarypox vectored vaccine provide longer duration of immunity, they sufficiently protect the equine population during an annual period between booster doses (Minke et al., 2007). Paillot et al. (2006a) studied the stimulation of the immune system after immunization with canarypox-based vaccine and subsequent challenge to a nebulized aerosol of EIV. Presence of humoral response was evidenced by serum antibody level and cell mediated response that was measured by production of IFN-γ. Post-challenge, the clinical signs were reduced with increased IFN-γ protein synthesis in vaccinated ponies. Fougerolle et al. (2016) demonstrated that all horses did not develop protective immunity after vaccination, resulting in an increased risk of infection and transmission. A field study was conducted to understand the poor response to primary EI immunization. During a study, 174 foals in 3 stud farms were immunized with canarypox-based vaccine and detectable antibody titer was observed after 2nd vaccination. After 3rd vaccination, there were still 19.2% poor antibody responders. The study proved its importance in evaluating herd immunity and its role in implementing correct vaccination management. Vaccination schedule would be different for different groups of equids. Vaccinating the breeding horse in later stages of pregnancy improved the antibody titer in colostrum. Foals are vaccinated routinely at the age of 6 months when the maternal antibody level decreases. Foals from unvaccinated dams need to be vaccinated early in their life (Daly et al., 2004; Heldens et al., 2007).

#### Modified vaccinia ankara vector (MVA)

The vector carrying HA and NP gene elicited good antibody response and also IFN-γ and mRNA production (Breathnach et al., 2004). Two of the recombinant modified vaccinia Ankara vector constructs with HA or NP genes were found to protect the ponies from clinical disease very effectively whereas vaccine with HA gene was found better than NP gene in terms of the protective outcome (Breathnach et al., 2006).

#### Modified herpes virus-1 vectored vaccine

Equine herpes virus-1 vectored vaccine carrying H3 gene of EIV generated robust protective immune response against influenza (VA05 and NY-99 strains) in horses (Van de Walle et al., 2010). This vaccine was found to be significantly more effective in terms of reduced viral shedding and mild clinical symptoms during the 1997 outbreak of EIV in Australia (Paillot and El-Hage, 2016). In addition, during this outbreak in 1997, both the ISCOM matrix based and canarypox vectored EI vaccine were found to be equally efficient in preventing clinical disease (Bryant et al., 2010).

#### Reverse genetics based vaccines

For influenza viruses including EIV, the field of reverse genetics (plasmid based) allows expression of the components of the virus involved in replication of the viral genome and transcription of gene (Nogales and Martínez-Sobrido, 2017). Live virus vaccine has been developed by reverse genetics technique employing HA and NA gene of eq/GA/81 wild-type (wt) virus along with the six internal protein genes of the ca A/Ann Arbor/6/60 (H2N2) vaccine donor virus, which form the base of seasonal live attenuated influenza vaccine licensed in the market. This vaccine was found to provide protection in mice and ferrets after heterologous challenge with H3N8 (eq/Newmarket/03) wt virus (Baz et al., 2015). Another vaccine generated by reverse genetics technology is a novel reassortant of ca strain A/HK/Otar/6:2/2010 containing HA and NA genes from wild-type strain A/equine/Otar/764/2007 (H3N8) and internal genes from ca A/Hong Kong/1/68/162/35CA (H3N2) strain in the form of nasal spray. The vaccine was found safe for intranasal administration in both yearlings and pregnant mares and it replicated exclusively in upper respiratory tract and did not lead to generalized infection (Tabynov et al., 2014). Carboxy-terminally truncated NS1 proteins are incapable of inhibiting type 1 IFN production by cells and are replication attenuated and thus are a vaccine candidate. Mutation at 126th amino acid position of NS1 protein, and subsequent aerosol or intranasal inoculation did not produce pyrexia with fewer clinical signs of illness and decreased virus shedding upon challenge (Chambers et al., 2009). Very recently, based on the reverse genetics vaccine approach, a temperature sensitive H3N8 EIV vaccine was developed that showed better protection both in mice and also in horse when challenged with wild type virus. As this mutant was developed by reverse genetics approach so it is possible to upgrade the vaccine strain whenever there is a mutation in the circulating virus and thus making it feasible to control the outbreak (Rodriguez et al., 2018).

#### DNA vaccines

DNA vaccines delivered through gene gun have been suggested for EIV (Lunn et al., 1999; Olsen, 2000; Dhama et al., 2008). DNA vaccines carrying HA gene elicited good cell mediated and humoral immunity eliciting IgG response, but it does not provoke IgA response (Soboll et al., 2003). These DNA vaccines based on H3N8 virus are quite safe and effective in eliciting both homologous and heterologous immune response (Ault et al., 2012). For the purpose of DNA vaccination, intra-lymphatic immunotherapy (ILIT) is the recent strategy, into which HA encoding plasmid is being injected in the sub-mandibular lymph node on days 0, 28, and 98 and such vaccination induced EIV specific immune response comparable to immune response evoked after natural infection, but lower than the conventional canarypox-based EIV vaccine (Landolt et al., 2010). Intranodal immunization allows vaccine delivery directly at the site of B and T lymphocytes priming, so improved immunity is expected. However, practical feasibility of this technique in the field is questionable due to the skills required for sub-mandibular injection with the risk of inaccurate injection in lymph node (Paillot, 2014).

After reviewing all existing vaccines, Consultative Committee for Emergency Animal Diseases (CCEAD) recommended to prefer a recombinant (canarypox) vectored vaccine owing to its ability to readily evoke efficient immune response against both the American and European lineage viruses (Perkins et al., 2011). Paillot et al. (2013b) have conducted experiments to reveal the pattern of EIV shedding after infection in vaccinated horses. The information turned out to be helpful regarding changes to current quarantine requirements. The findings showed that the viral shedding detection is better in nasopharyngeal swabs than nasal swab in terms of frequency and amount of virus. Recently a study reported the use of individual nebulization for challenge infection in equines as the present method of the use of room nebulization showed decreased pathogenicity after challenge. The authors also suggested that the use of individual nebulization can decrease the use of animal numbers in a group, thus follows the 3R's principle (Replacement, Reduction, and Refinement) (Garrett et al., 2017).

**Figure 5:**
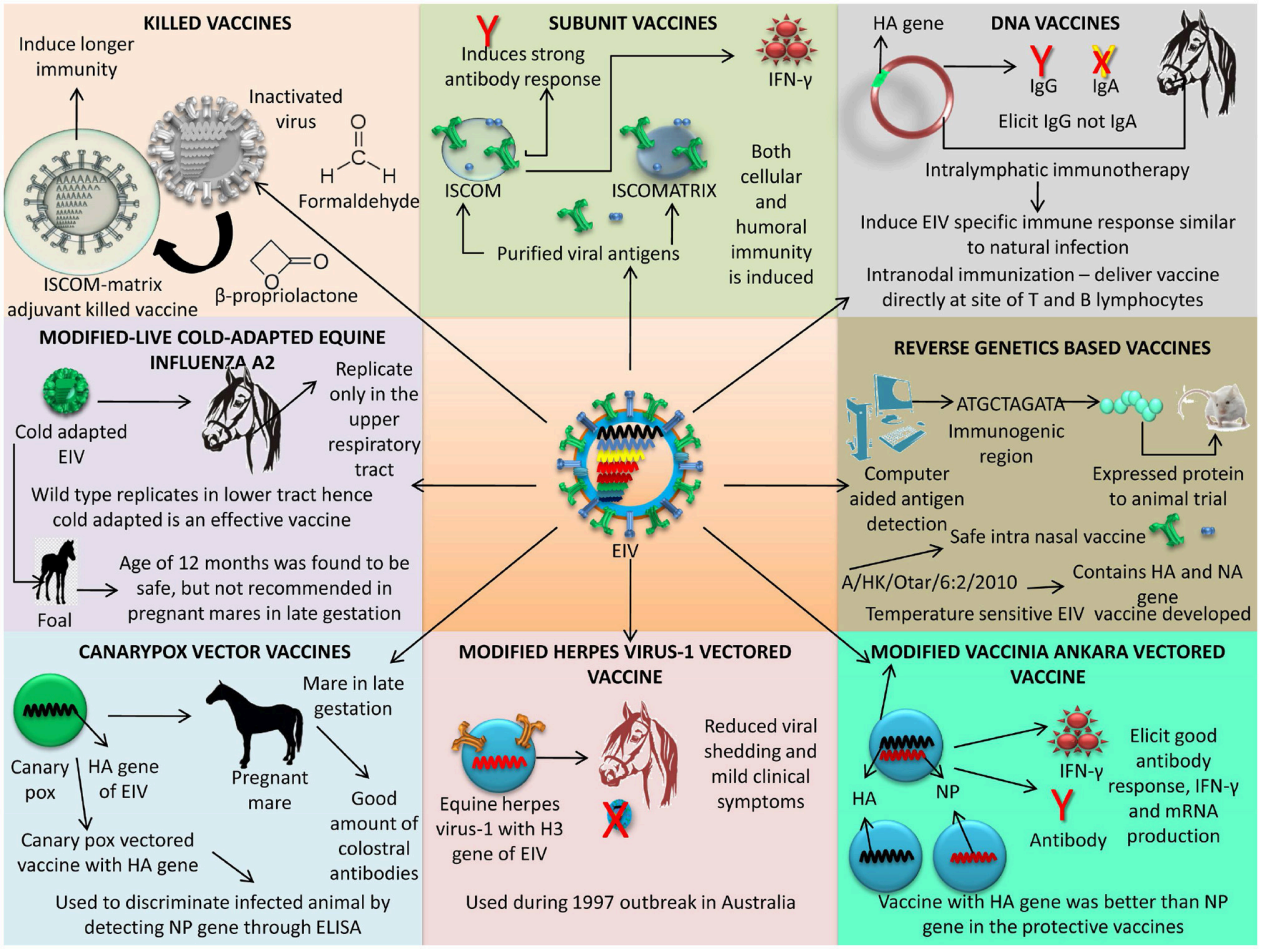
Different vaccine platforms available for EIV. Platforms include killed vaccine, inactivated vaccine, subunit vaccine, DNA vaccine, subunit vaccine, vectored vaccine, reverse genetics-based vaccine.

Different vaccine platforms available for prevention of EIV infection are presented in Table 1, Figure 5.

**Table 1:** The account of vaccination strategies in-use for Equine influenza worldwide.

**S. No**.	**Vaccine type**	**Specific note**	**Study outcome**	**Antigen used in vaccine**	**Name of vaccine**	**Model organism**	**Reference(s)**
1.	Killed vaccine adjuvanted with ISCOM-matrix	Strong antibody response with elevated levels of IFN-γ	ISCOMatrix second generation adjuvant helps to raise primary antibody titer to a value that is sufficient to prevent infection until the time of annual revaccination 12 months later	Purified HA and NA proteins of equine influenza virus strain A/equi-1/Prague/56(H7N7), A/equi2/Newmarket-1/93 (H3N8-American type strain) and A/equi-2/Newmarket-2/93 (H3N8-European type strain) + tetanus toxoi.	Prequenza Te	Fjord horse (*n* = 12)	Heldens et al., 2009
			A significant reduction of virus in excreta and reduction in virus induced pyrexia				
2.	ISCOM-based EI vaccine	Vaccinated and infected animals may be a source of infection by EIV shedding	Vaccinated and infected horse may shed virus and able to infect commingling sentinels. The virus shedding by comminglings is detectable upto 6 days after commingling	Antigens from the strains A/eq1/Newmarket/77 (H7N7), A/eq/Borlange/91 (H3N8, European lineage) and A/eq/Kentucky/98 (H3N8, American lineage).	Equip™ FT	Pony (*n* = 7)	Paillot et al., 2013b
3.	Cold adapted and pox-vectored	Mimic natural infection by generating both humoral and cellular response	Vaccinated ponies produced high amount of anti-influenza virus IgGa and IgGb antibodies and in statistically significant manner protected from clinical signs of disease	Two live recombinant canarypox viruses expressing the HA of A/eq/KY/94 (American lineage) and A/eq/NM/2/93 (Eurasian lineage), respectively	RECOMBITEK	Ponies (*n* = 43)	Soboll et al., 2010
4.	Inactivated equine influenza virus vaccine + equine herpes virus	EIV and EHV-1/4 combination increased antibody response to EIV and did not compromise the humoral immune response to EHV-1/4.	After booster dose there was no significant difference in antibody titer between the EIV only vaccine or EIV + Herpes virus vaccine	A/eq/1/Prague/56 (H7N7) + A/eq/Suffolk/89 (H3N8-European lineage) + A/eq/Newmarket/1/93 (H3N8-American lineage) + inactivated EHV-1 strain 438/77 and EHV-4 strain 405/76.	EIV vaccine Duvaxyn IE + bivalent Duvaxyn EHV-1,4	Horse (*n* = 30)	Gildea et al., 2016
5.	Modified-live cold-adapted A2 strain (Intranasal immunization)	No reversion to virulence strain	The vaccine provided protection form clinical EIV infection caused by Equine-2 influenza viruses (American lineage) and A/equine-2/Saskatoon/90 (Eurasian' lineage)	A/eq/Kentucky/1/91 (H3N8)	Flu Avert IN	Ponies (*n* = 16)	Chambers et al., 2001; Townsend et al., 2001
6.	Canary pox vectored vaccines (Intranasal immunization)	Generate colostral antibodies so used during gestation period. The recombinant virus cause an abortive infection in mammalian cells, therefore no progeny viruses are made however viral proteins are expressed, processed endogenously and presented by MHC class I molecules	Meets OIE recommendations for updated EI vaccine and significant protection in vaccinated ponies was observed in comparison to control	A/eq/Richmond/1/07 isolate (Florida clade 2 sub-lineage) + A/eq/Ohio/03 (Florida clade 1 sub-lineage), vectored with canarypox	ProteqFlu	Pony (*n* = 14)	Paillot et al., 2014
7.	Equine herpes virus-1 based live vaccine (Carrying H3 gene)	Robust protective immunity against VA05 and NY-99 strains	Elicited a long lasting serological response against both EHV-1 and EIV	Codon-optimized H3 sequence from A/equine/OH/03	—	Horse (*n* = 12)	Van de Walle et al., 2010
8.	Live attenuated reverse genetics based vaccine	A single dose of the vaccine was highly immunogenic and efficacious against wild type virus challenge	Prevent from heterologous challenge	Virus backbone from cold adapted strain A/Ann Arbor/6/60 and HA and NA genes from eq/GA/81 (H3N8)	—	Ferrets and Mice (*n* = not provided in manuscript)	Baz et al., 2015
9.	DNA vaccines (containing HA gene)	Cellular and humoral immunity with IgG production, but not IgA	Generation of virus-specific IgGa, IgGb and IFN-γ responses	HA gene of A/Equine/Kentucky/1/81 (Eq/Ky) cloned in plasmid WRG7077. Plasmid construct expressing IL6 was also co vaccinated	—	Pony (*n* = 25)	Soboll et al., 2003
10.	Modified vaccinia Ankara vector (MVA) containing HA + NP	Influenza virus-specific lymphoproliferative responses and IFN-γ production against HA and NP	DNA prime-MVA boost vaccination indicated the protective efficacy	HA and NA genes of A/equine/Kentucky/1/81 (Eq/Ky) cloned in an MVA construction plasmid	—	Pony (*n* = 4)	Breathnach et al., 2004

### Biosecurity

Apart from vaccination, other strategies that contribute toward the control of EI include follow up of strict biosecurity measures, restricted movement and traffic, proper quarantine practices, and post vaccination surveillance programs. Importance of implementing quarantine practices strictly can be understood from the experience of the 2007 outbreak in Australia that occurred due to negligence at the time of importation of horses (Watson et al., 2011). In New Zealand, on experimental basis, InterSpread Plus (stochastic simulation model) was used in order to evaluate the efficacy of control programs. Out of various strategies evaluated for control of this disease, vaccination combined with complete movement restriction was found to be very effective in this direction in New Zealand (Rosanowski et al., 2016).

Biosecurity measures and appropriate management practices are essential for prevention and control of EI. In the event of outbreak, adoption of biosecurity measures can provide protection to horses (Schemann et al., 2012). Simple preventive measures based on personal hygiene, decontamination, and other biosecurity practices prevented spread of infection in Queensland, Australia (Frazer et al., 2011). A protocol of hygiene practices devised by a practice in which all veterinarians were involved on a daily basis in visiting infected premises, including sampling, handling of equines, treatment of clinical cases, and other common veterinary work has been described, which should be strictly followed in face of an equine influenza outbreak (Major, 2011). The implementation of strategy like high health, high performance (HHP) by OIE provides the mitigation measures for mitigating the spread of equine diseases including EI (Dominguez et al., 2015).

There are various factors and many different players responsible for prevention and control of EI in equine sports arena viz. vaccine producers, vaccine regulators, OIE, various government bodies in different countries, veterinary practitioners, owners, riders, trainers, etc. (Cooke, 2013). Each has a unique and important role to play in proper management of equestrian sports (Cooke, 2013). A cross sectional study was conducted on 759 Australian horse owners to determine their biosecurity practices and perceptions. It demonstrated that the young aged people, or people having no commercial involvement with horses or having no business impact of EI outbreak, were likely to have poor biosecurity compliance (Arthur and Suann, 2011).

### Treatment

Mainly the managemental practices should be followed in animals affected with EIV. As a general protocol horses should be rested for the number of days/weeks equal to the number of days/weeks they had suffered fever which can aid in recovery of the respiratory epithelium (Chambers et al., 1995). Gradual work can be provided after complete rest as sudden work load can lead to chronic obstructive pulmonary disease and mycocarditis. Free air flow should be present in the stall during the resting period so that good quality oxygen supply will be available. Good hygienic food, water and dust free bedding materials should be provided at the stall (Chambers et al., 1995). Though amantadine has been tried, there are no specific antiviral drugs available in the market for the treatment of EIV (Radostits et al., 2003). Treatment is mainly symptomatic and antibiotics like penicillin G or trimethoprim/sulfonamide are administered to prevent from secondary bacterial infections due to *Actinobacillus* species and *Streptococcus* species. Non-steroidal anti-inflammatory drugs like phenylbutazone, flunixin meglumine, or dipyrone can be administered to reduce fever (Wilson, 1993). Neuraminidase inhibitors such as peramivir at the early stage of infection are recommended. This drug was found to reduce virus shedding and as such limits the spread of infection from one horse to another (Yamanaka et al., 2015). Peramivir is a selective NA inhibitor having strong affinity for NA and with a slow off-rate of NA from NA-peramivir complex, providing a prolonged inhibitory effect and subsequent lower dose requirement. Single intra-venous dose of peramivir (7.8–9.3 mg/kg of body weight) exhibited significant reduction in pyrexia, nasal discharge and cough and also in duration of viral shedding. It indicated the great potential of peramivir as treatment of equine influenza and also in line able to contain the spread of the disease (Yamanaka et al., 2012b; Cullinane and Newton, 2013).

Few of the promising anti-viral therapeutic regimens such as the use of cytokines, microRNA, si-RNA, TLRs, potent immunomodulators, nanotechnology based therapeutics, herbal and plant metabolites, and others which might have feasibilities to counter EIV needs to exploited optimally (Blecher et al., 2011; Dhama et al., 2013, 2014, 2018; Malik et al., 2013; Junquera et al., 2014; Prasad et al., 2018).

## Conclusion and future prospects

Equine influenza, a highly contagious respiratory disease of horses mainly caused by H3N8, is a major problem globally. There are many EIV subtypes circulating and which provide no cross-protection to other strains. Recurrent epidemics lead to reduction in horse performance. The EI outbreaks have regularly been witnessed in non-vaccinated as well as vaccinated herds and thus this disease has acquired a serious issue worldwide. Continuous emergence of newer strains due to mutations is yet another hindrance to the definite solution of this disease by means of vaccination. Though, no human cases in practice have been noticed yet it should be realized that under experimental settings this virus has shown the ability to infect humans with antibody induction. Of note, this virus has crossed the species barrier by causing disease in various other species (dogs, cats, camels). This should raise alarm as dogs are closely associated with humans and they may act as a mixing vessel for equine and human influenza virus facilitating emergence of new human influenza strains. It is imperative now to develop an efficient canine influenza virus vaccine and imply proper vaccination strategy in dogs to prevent such infections.

Recent advances in diagnosis and surveillance of EIV need to be exploited to their full potential to monitor the epidemiology of this virus in details and recording incidences and disease outbreaks of EI. Regular monitoring of equine population for EIV strains is mandatory to transpire the effective vaccination strategies. Better methods for assessing vaccine potency also need to be designed. A thorough understanding of disease pathogenesis will help in selecting protective epitopes for vaccine designing. Regular monitoring and surveillance of the virus can also help to update the available vaccine with the new variants that occur as due course of time owing to the evolution of the virus. Such regular updation of vaccine and also testing the potency and efficacy of the updated vaccine are also mandatory to prevent and protect equine population from EIV keeping in view the evolution of the virus. It is the right time to opt various types of advancements in vaccine manufacturing and adjuvants technology for developing safe and effective EIV vaccines. Also, vaccine delivery methods can be standardized for superior quality of immune response. Further improvements in assays monitoring EIV specific antibody levels in the serum can be done, which better correlate with protection status.

## Author contributions

All the authors substantially contributed to the conception, design, checking and approving final version of the manuscript, and agree to be accountable for its contents. RKS and KD initiated this review compilation. RK, KK, AM, SK, and SC updated various sections. NV reviewed Indian Scenario. MM and YM reviewed. RK designed tables and KK designed the figures. JK, RKS, RS, BT, and KD overviewed, thoroughly checked, and finally edited the whole manuscript.

### Conflict of interest statement

The authors declare that the research was conducted in the absence of any commercial or financial relationships that could be construed as a potential conflict of interest.
